# Antibiotic-Resistant Bacteria in Greywater and Greywater-Irrigated Soils

**DOI:** 10.3389/fmicb.2018.02666

**Published:** 2018-11-06

**Authors:** Eleonora Troiano, Luciano Beneduce, Amit Gross, Zeev Ronen

**Affiliations:** ^1^Department of the Sciences of Agriculture, Food and Environment, University of Foggia, Foggia, Italy; ^2^Department of Environmental Hydrology and Microbiology, Zuckerberg Institute for Water Research, Jacob Blaustein Institutes for Desert Research, Ben-Gurion University of the Negev, Midreshet Ben-Gurion, Israel

**Keywords:** greywater, antibiotic resistance, tetracycline, irrigation, recirculating vertical flow constructed wetland

## Abstract

This study represents the first systematic attempt to evaluate antibiotic-resistant bacteria (ARB) occurrence in treated greywater and the potential spread of these bacteria from the greywater to greywater-irrigated soil. Treated greywater from three recirculating vertical flow constructed wetlands, each located in a household in the central Negev Desert, Israel, was surveyed. The presence of antibiotic-resistant bacteria in raw and treated greywater was investigated with culture and molecular methods, as well as their presence in the corresponding treated-greywater-irrigated soils. Additionally, the effectiveness of chlorination to prevent the spread of ARB was tested. The total count of tetracycline-resistant bacteria significantly increased in the treated greywater, likely due to their concentration on the filter matrix of the treatment systems. Twenty-four strains of tetracycline-resistant bacteria were isolated and identified at the genus level by 16Sr RNA gene sequencing. All the tetracycline-resistant bacteria showed high resistance traits, and some of them presented multiple antibiotic resistances. Six tetracycline resistance genes (coding for efflux and ribosomal resistance mechanisms) and five β-lactamase genes were detected. In 14 of the isolated strains, the gene *tet*39, which is phylogenetically related to both environmental and clinical strains, was identified. All the *tet*39 resistant bacteria were positive to at least one of the β-lactamase genes tested. Chlorination was found to be an efficient method to reduce ARB in treated greywater. We concluded that disinfection of treated greywater may reduce the risks not only from the potential presence of pathogens but also from the presence of ARB and antibiotic resistance genes.

## Introduction

The modern lifestyle requires a large quantity of potable water and generates large amounts of wastewater (Eriksson et al., 2002; Schacht et al., 2016). This, in combination with dwindling water resources worldwide, has led to increasing interest in wastewater reuse in many parts of the world, including both industrialized and developing countries (Eriksson et al., 2002).

One method of conserving water, on the local scale, is by recycling greywater (GW) for irrigation (Gross et al., 2007). Greywater is defined as domestic wastewater that excludes wastewater from toilets and typically includes water from baths, showers, hand basins, and washing machines (Jefferson et al., 2000; Gross et al., 2007; Ghaitidak and Yadav, 2015). Greywater constitutes 50–80% of the total household wastewater, and its recycling can reduce potable water use by up to 50% (Gross et al., 2007). In recent years, there has been an increase in the use of GW for various purposes such as toilet flushing, landscaping, and garden irrigation (Gross et al., 2015).

It has been well-established that raw GW is contaminated with pathogens (although less than “full” domestic wastewater) and other chemical contaminants and thus should be treated before reuse (James et al., 2016). Potential health risks associated with the spread of pathogenic organisms through the use of treated GW are critical issues (Benami et al., 2016). In fact, a number of pathogens are occasionally found in raw GW (RGW), including fecal coliforms, fecal enterococci, fecal streptococci, *Klebsiella pneumoniae*, and *Pseudomonas aeruginosa*, among others (Benami et al., 2016). Interestingly contradicting results regarding increasing levels of fecal coliforms in soils following long term greywater irrigation were reported (Casanova et al., 2001; Benami et al., 2016). While Casanova reported on significant increase in fecal coliforms, Benami et al. (2013) reported no such differences.

Another source of recent concern is the spread of antibiotic-resistant bacteria from GW, as well as the evolution and propagation of antibiotic-resistant microorganisms (Rizzo et al., 2013; Berendonk et al., 2015). The intensive use of antibiotics for human medical, veterinary, and agricultural purposes results in their continuous release into the environment (Rizzo et al., 2013), with the primary concern of the development of antibiotic resistance genes (ARGs) and antibiotic-resistant bacteria (ARB), which reduce the therapeutic potential against human and animal pathogens (Kemper, 2008; Zhang X. X. et al., 2009).

The presence of ARB and ARGs, even at very low levels in the household garden, may represent a high risk to human health through the spread of antibiotic resistance, especially if humans have high exposure to places where ARB are present (e.g., food crops cultivated in GW-irrigated fields). ARGs may persist in the environment, and even worse, they can be spread to other bacteria including human commensals or pathogens of clinical relevance, through the horizontal gene transfer (HGT) of mobile genetic elements (Christou et al., 2017).

The dissemination of ARB and ARGs is an alarming problem because it has been demonstrated that intrinsic antibiotic resistance might have been selected in the course of bacterial evolution, even without antibiotic selective pressure, for covering functions other than antibiotic resistance (Alonso et al., 2001). For example, it was shown that non-antibiotic biocidal compounds such as triclosan in greywater increase the prevalence of ARB in the soil microcosm (Harrow et al., 2011).

Nevertheless, recent studies demonstrated that irrigation with treated municipal wastewater does not seem to impact antibiotic resistance levels in the soil microbiome (Gatica and Cytryn, 2013). Thus, our initial hypothesis was that greywater doesn't harbor ARB and that treated GW will not increase the abundance of ARB in TGW irrigated soil. However, there is still a lack of evidence about the potential efficacy of actual GW treatment before reuse on ARB abundance and the potential contribution of GW irrigation to the spread of ARB. Understanding the dynamics of ARB and ARGs in the urban water cycle is an increasingly important goal as antibiotic resistance is recognized as one of the most significant human health challenges of the Twenty-first century (WHO, 2012; Voolaid et al., 2018).

Therefore, the objectives of this work were to investigate the prevalence of ARB and ARGs in raw and biologically treated GW, as well as their presence in the corresponding treated-GW-irrigated soils. We also tested the effect of chlorination on the survival of ARB. Specifically, we focused on tetracycline-resistant bacteria because tetracyclines were the first primary group to which the term “broad spectrum” was applied. For their spectrum of activity, their relative safety, and their low cost, tetracyclines have been used widely across the globe for clinical and non-clinical uses and, are the fifth most consumed antibiotics in the world (Van Boeckel et al., 2014). Furthermore, tetracycline resistance bacteria are widespread in treated wastewater from Israel leading us to believe that they present also in greywater (Gatica and Cytryn, 2013).

## Materials and methods

### Location and sampling

Raw and treated GW from three different households in the central Negev Desert, Israel (30°51′05″ N 34°47′00″ E) were monitored. GW treatment was done by a recirculating vertical flow constructed wetland (RVFCW) as described by Gross et al. (2007) and the system layout and operation parameters are presented in Figure S1. The three systems were selected since they have been working now for over 7 years and the treated greywater TGW is used continuously for irrigation at this time in parallel to freshwater irrigated controls. All households contain kids of different ages. TGW samples were collected routinely and analyzed for physicochemical parameters by standard methods (Table S1) as well as ARB and ARGs (as described below). From each household, 100 mL of water (raw and treated) were taken and placed in two sterile 50-mL falcon plastic tubes. Ten tuff gravel pieces with an average weight of 8 g were taken from the upper surface layer of the RVFCW bed for ARB and ARG biofilm analyses. Similarly, ARB and ARGs were monitored in freshwater- and treated-GW-irrigated soils. Duplicate soil samples from each location (15 g of soil at 5 cm depth) were collected twice, in January and March 2017. All samples were immediately transported to the laboratory and analyzed within a few hours.

### Isolation and count of tetracycline-resistant bacteria from water, filter bed, and soil

A modified PTYG broth (peptone, tryptone, yeast extract, glucose) was used at 10% of the original strength and without sodium thioglycollate (Atlas, 2010). The PTYG media were solidified by using 15 g L^−1^ of bacteriological agar (Difco, Franklin Lakes, NJ, USA). For bacterial extraction, 3 g of soil was suspended in 10 mL of the sterile PTYG broth and then shaken for 5 min on an orbital shaker at 200 RPM at 25°C. The solids settled for 5 min, and 100 μL of the supernatant was used to prepare the dilutions. The dry weight of the soil and the tuff gravel was obtained after drying for 24 h at 65°C. The supernatant from this slurry was used for dilutions, counting, and microbial isolation.

For the isolation of tetracycline-resistant bacteria, 0.1 mL of the serial dilutions of the different samples [raw greywater (RGW), biofilm (BF), treated greywater (TGW), greywater-irrigated soil (TS) and freshwater-irrigated soil (US)] was spread in duplicate with a sterile disposable Drigalski spreader on the agar surface of two different types of plates: the control containing only the medium PTYG and the second containing PTYG + tetracycline (20 mg L^−1^ Sigma Park Rabin, Rehovot, Israel). The CLSI guidelines (2014) were used as a benchmark for isolating tetracycline-resistant bacteria. Accordingly, isolates with MIC values of Tetracycline at ≥ 16 μg mL^−1^ are regarded as resistant, and thus we applied a concentration of 20 μg mL^−1^ in our isolation plates.

For both the control and the treated samples of water, soil, and biofilm, suitably diluted samples were inoculated in the respective plates and were incubated at 25°C for 48 h and at 37°C for 24 h. After the incubation period, the colonies were counted. The percentage of tetracycline-resistant bacteria was obtained from the ratio between the colony count on the plates containing tetracycline and the colony count on the control plates.

From the isolation plate containing tetracycline, colonies with distinct morphologies were taken with a sterile loop and streaked on a fresh plate of PTYG + tetracycline (20 mg L^−1^) and cycloheximide (20 mg L^−1^ Sigma Park Rabin, Rehovot, Israel). After an incubation period of 48 h for the bacteria incubated at 25°C and 24 h for the bacteria incubated at 37 °C, all strains were purified by streaking them twice on a fresh sterile plate of PTYG + antibiotic. The isolates were stored at −80°C in glycerol (25%), PTYG, and tetracycline (20 mg L^−1^).

### Tap water analysis

To confirm that the tetracycline-resistant bacteria did not originate from the tap water, 0.1-mL samples of tap water, collected from the three different households, were spread on the surface of the agar plate with or without tetracycline (20 mg L^−1^).

### Identification of tetracycline-resistant bacteria

The isolated tetracycline-resistant bacteria (*n* = 24) were identified at the genus level by 16S rRNA gene sequencing by Hy Laboratories Ltd. (Rehovot, Israel). Following DNA isolation, the first ~800 bp region of the 16S rRNA gene was amplified by PCR, and the resulting amplicon was sequenced using an ABI3730xl genetic analyzer and BigDye V1.1 chemistry, according to the manufacturer's instructions. The obtained sequence was analyzed using sequencing analysis software (Applied Biosystems v5.4) and compared with archived NCBI sequences for gene identification. Sequences of 16S rRNA genes were deposited in Genbank with accession numbers from MH090940 to MH090963. Nucleotide sequences were aligned and compared, and were then used to infer a phylogenetic tree with MEGA7.0.14 (Kumar et al., 2016).

### The growth of tetracycline-resistant bacteria in the presence of chlorine

To evaluate the possible effect of chlorine on the viability of tetracycline-resistant strains, the growth of *Serratia spp*. strains, an opportunistic pathogen, isolated from SYS3, was examined in the absence (control group) and the presence of 2 mg L^−1^ of free chlorine as NaClO. The initial culture was about 1 × 10^6^ CFU mL^−1^ that was incubated in treated greywater at either 25°C or 37 °C. The suspension sampled was diluted hourly, and then 10 μL was spotted on the plate. The colonies in the spots were counted after 24 h of incubation under a magnifying glass.

### Multiple resistances

The isolated tetracycline-resistant bacteria were also evaluated for possible multiple resistances to three different antibiotics (all from Sigma): amoxicillin (β-lactams), ciprofloxacin (fluoroquinolones), and kanamycin (aminoglycosides). The bacteria were streaked on PTYG agar plates containing 20 mg L^−1^ of each one of the three antibiotics. The plate was incubated at 25°C (for the bacteria isolated at 25°C) and 37°C (for the bacteria isolated at 37 °C).

### Minimum inhibitory concentration (MIC)

For all the isolated tetracycline-resistant bacteria, the MIC of tetracycline was tested based on the broth microdilution protocol (Wiegand et al., 2008). In addition, the isolates that were shown to be also able to grow in the presence of 20 mg L^−1^ of amoxicillin, ciprofloxacin and kanamycin were tested for the MICs of these three antibiotics. Filtered (0.22 μm) stock solutions of antibiotics (0.5 mg/ml) were dissolved in distilled water. Strains from glycerol stocks were inoculated in PTYG and incubated overnight. After 12 h, the optical density (OD) of the samples was measured, and the bacterial cultures were diluted to an OD of 0.1 (corresponding to about 5.7 × 10^7^ CFU mL^−1^), and then 50 μL was used for MIC determination.

The tetracycline MIC was tested at concentrations ranging from 100 to 350 μg mL^−1^ for the bacteria isolated at 37°C and from 100 to 500 μg mL^−1^ ml for the bacteria isolated at 25 °C. The other three antibiotics were tested at concentrations ranging from 50 to 300 μg mL^−1^. For the experiment, multiple sterile 48-well plates (Costar, Corning, NY, USA) were used. In each plate, the wells of the first column were used as a negative control and contained only 500 μl of the PTYG medium; the wells of the second column were used as a positive control and contained 450 μL of PTYG and 50 μL of the tested strain; the remaining wells were used as a test group and contained 450 μL of PTYG to which was added six different antibiotic concentrations. The test was performed in duplicate. The OD of 600 nm at time zero and after 12 h was measured with a multi-plate reader (Infinite^®;^ 200 PRO, Tecan Männedorf, Switzerland). The % inhibition of all samples was calculated, using the following formula:

$$\begin{array}{l} \%\;inhibition=\frac{OD\;positive\;control-OD\;given\;concentration}{OD\;positive\;control-OD\;negative\;cobtrol}\times 100 \end{array}$$

To determine the MIC value (μg mL^−1^), the following criterion was used: between wells with no bacterial growth, the one with the lowest antibiotic concentration indicates the MIC value. The results were reported in the following way: the values preceded by the sign ≤ indicate that the microorganism growth was inhibited by the lowest concentration of the antibiotic used for the test, while values preceded by the sign ≥ indicate that growth was not inhibited by the higher concentrations of the antibiotic tested.

### DNA and plasmid extraction

Nucleic acid extraction from an overnight culture of each strain in PTYG plus tetracycline (20 mg L^−1^) was performed using a GenElute Bacterial Genomic DNA kit (Sigma) following the manufacturer's protocol. The concentration and quality of the DNA were determined by spectrophotometric analysis and agarose gel electrophoresis. For the spectrophotometric analysis, the NanoDrop® ND-1000 (NanoDrop Technologies, Wilmington, DE, USA*)* was used. Electrophoresis visualization of DNA was performed on 0.8% of agarose stained with Gel Red (Biotium, Fremont, CA, USA).

Positive controls of β-lactamase genes were cloned in different plasmids. The blaOXA2 and 10 were synthesized and cloned by Syntezza Bioscience Ltd. (Jerusalem, Israel) on a vector pUC57 (Rocha et al., 2018) provided by Dr. Eddie Cytryn (The Institute of Soil, Water, and Environmental Science, Volcani Center, Israel); the CTX- M32 and blaTEM genes were cloned on a pNORM kindly provided by Christophe Merlin (the University of Lorraine, Laboratory of Physical Chemistry and Microbiology for the Environment, Nancy, France); the blaSHV from the amoxicillin-resistant *K. pneumoniae* strain G-A-TGW (MG982455.1) was cloned in a pJET vector.

### PCR analyses

For the presence of β-lactamases, five genes were evaluated, including blaTEM, blaCTXM-32, and blaSHV that belong to the class A of β -lactamase, and blaOXA-2 and blaOXA-10 that belong to the class D of β-lactamase. For tetracycline, six genes were evaluated: *tet*39, *tet*A, and *tet*B (efflux), and *tet*M, *tet*Q, and *tet*W (ribosomal).The primers and sizes of the PCR products are presented in Table 1. The PCR conditions appear in the Table S2.

**Table 1:** PCR primers that were used in the work for screening of isolated strains.

**Target gene**	**Primer name**	**Sequence (5′-3′)**	**Amplicon size (bp)**	**References**
blaSHV	bla-SHV- F	CGCTTTCCCATGATGAGCACCTTT	110 bp	Xi et al., 2009
	bla-SHV-R	TCCTGCTGGCGATAGTGGATCTTT	
blaTEM	qblaTEM-F	TTCCTGTTTTTGCTCACCCAG	113 bp	Muyzer et al., 1993
	qblaTEM-R	CTCAAGGATCTTACCGCTGTTG	
blaCTX-M32	CTXM-F	CTATGGCACCACCAACGATA	156 bp	Bibbal et al., 2007
	CTXM-R	ACGGCTTTCTGCCTTAGGTT	
blaOXA-2	OXA-2 F	AAGAAACGCTACTCGCCTGC	478 bp	Bert et al., 2002
	OXA-2 R	CCACTCAACCCATCCTACCC	
blaOXA-10	OXA-10 F	TCAACAAATCGCCAGAGAAG	276 bp	Bert et al., 2002
	OXA-10 R	TCCCACACCAGAAAAACCA	
*tet*M	TetM-F	ACAGAAAGCTTATTATATAAC	171 bp	Aminov and Mackie, 2001
	TetM-R	TGGCGTGTCTATGATGTTCAC	
*tet*Q	TetQ-F	AGAATCTGCTGTTTGCCAGTG	169 bp	Aminov and Mackie, 2001
	TetQ-R	CGGAGTGTCAATGATATTGCA	
*tet*W	TetW-F	GAGAGCCTGCTATATGCCAGC	168 bp	Aminov and Mackie, 2001
	TetW-R	GGGCGTATCCACAATGTTAAC	
*tet*A	Tet A-F	GCGCGATCTGGTTCACTCG	164 bp	Aminov et al., 2002
	Tet A-R	AGTCGACAGYRGCGCCGGC	
*tet*B	Tet B- F	TTGGTTAGGGGCAAGTTTTG	659 bp	Fan et al., 2007
	TetB- R	GTAATGGGCCAATAACACCG	
*tet*39	tet(39)-F	CTCCTTCTCTATTGTGGCTA	701 bp	Adelowo and Fagade, 2009
	tet(39)-R	CACTAATACCTCTGGACATCA	

All the positive *tet*39 PCR products were purified and sequenced by Macrogen (Amsterdam, the Netherlands). Sequences of *tet*39 were deposited in Genbank with the accession numbers MH106412 to MH106425. Nucleotide sequences were aligned and compared, and then were used to infer a phylogenetic tree with MEGA7.0.14 (Kumar et al., 2016).

### Statistical analysis

The result of the bacterial count was plotted in histograms and box plots demonstrating means and standard deviation. The differences in the total bacterial count (TC) and the tetracycline-resistant bacterial count (TRBC) were compared by an analysis of variance (ANOVA) with *p* < 0.05 for significance, using Past 3.19 Software (Hammer et al., 2001).

## Results

### Tetracycline-resistant bacteria quantification and isolation

**Figure 1:**
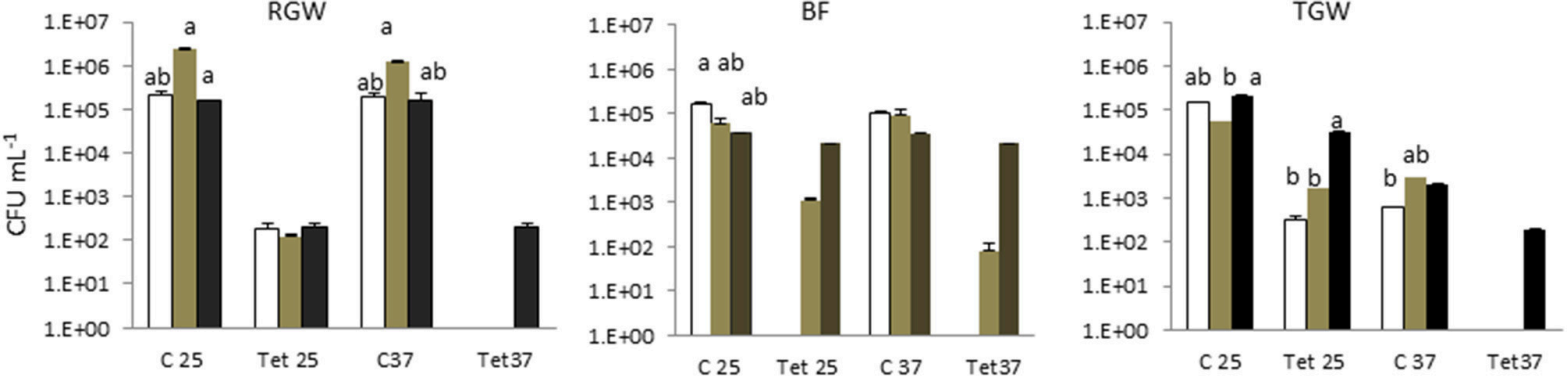
Total bacterial count (TBC) and tetracycline (tet)-resistant bacterial count (TRBC) in the three examined systems in all sampling locations. SYS1 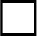, SYS 2 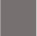, SYS 3 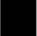. RGW (raw greywater); BF (biofilm); TGW (treated greywater). C25: total bacterial count (TC), incubation at 25°C. Tet25, tetracycline-resistant bacterial count (TRBC), incubation at 25°C; C37, TC, incubation at 25°C; Tet37, TRBC, incubation at 37°C. Error bars represent standard deviation of duplicate plate counts for each of the three examined systems in all sampling location. ^a^, ^ab^, ^b^ superscript letters indicate significant differences (*P* < 0.05) between the three systems.

Tetracycline-resistant bacteria were not isolated in tap water in any of the households. This study showed that the two isolation temperatures did not cause a significant difference (*p* > 0.05) in the total bacterial count in the different sampling locations (water, biofilm, and soil), but they did play a significant role (*p* < 0.05) in the tetracycline-resistant bacterial count (TRBC). The total bacterial count and the TRBC in all three examined systems in all sampling locations are presented in Figure 1. The total bacterial count in the RGW level of SYS1 was about an order of magnitude higher than in the other systems at both incubation temperatures. By contrast, the TRBC was lower and no significant differences (*p* > 0.05) were detected at the 25°C isolation temperature for the three households, while only for the SYS3 system was a detectable level of tetracycline-resistant bacteria found at 37°C.

**Figure 2:**
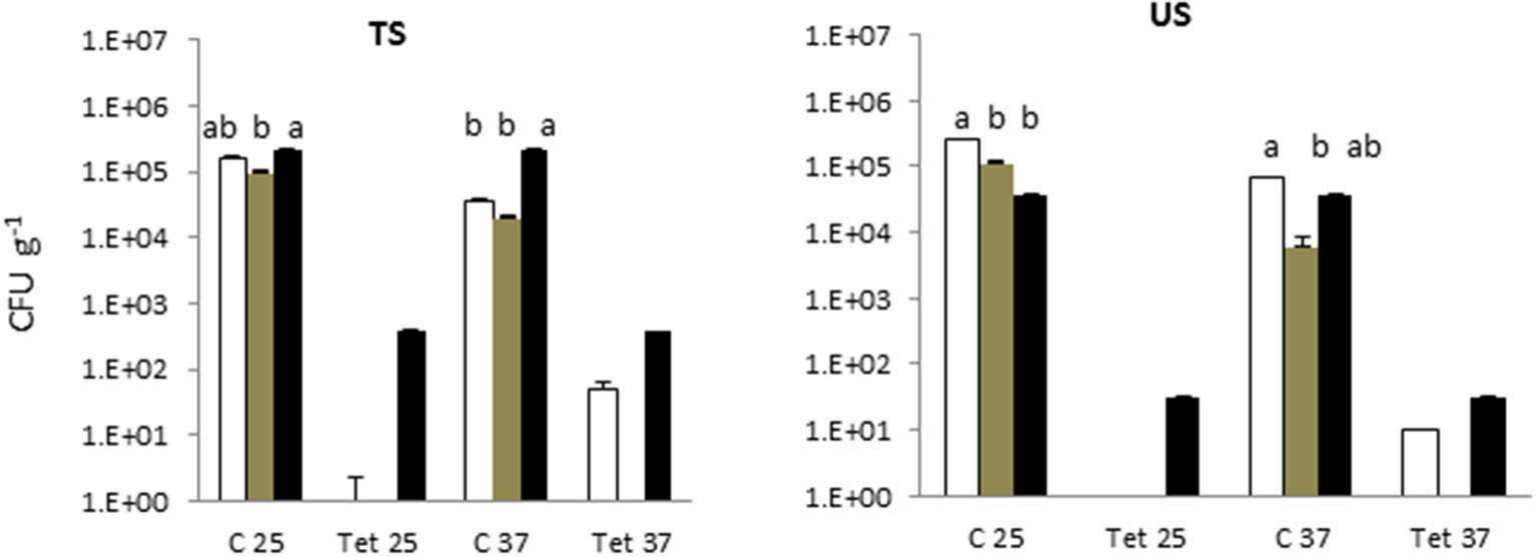
Total bacterial count (TBC) and tetracycline (tet)-resistant bacterial count (TRBC) in the in the soils of the three examined systems in all sampling locations. SYS1 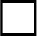, SYS 2 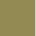, SYS 3 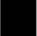. TS (greywater irrigated soil); US (freshwater-irrigated soil). C25, total bacterial count (TC), incubation at 25°C. Tet25, tetracycline-resistant bacterial count (TRBC), incubation at 25°C; C37, TC, incubation at 25°C; Tet37, TRBC, incubation at 37°C. Error bars represent standard deviation of duplicate plate counts for each of the three examined systems in all sampling location. ^a^, ^ab^, ^b^ superscript letters indicate significant differences (*P* < 0.05) between the three systems.

In the soil irrigated with freshwater and with treated greywater, the total microbial counts at 25°C and 37°C were 5 Log CFU g^−1^ (Figure 2). Detectable levels of tetracycline-resistant bacteria were found only in the SYS2 (at 25°C) and SYS3 (at both incubation temperatures) systems.

**Figure 3:**
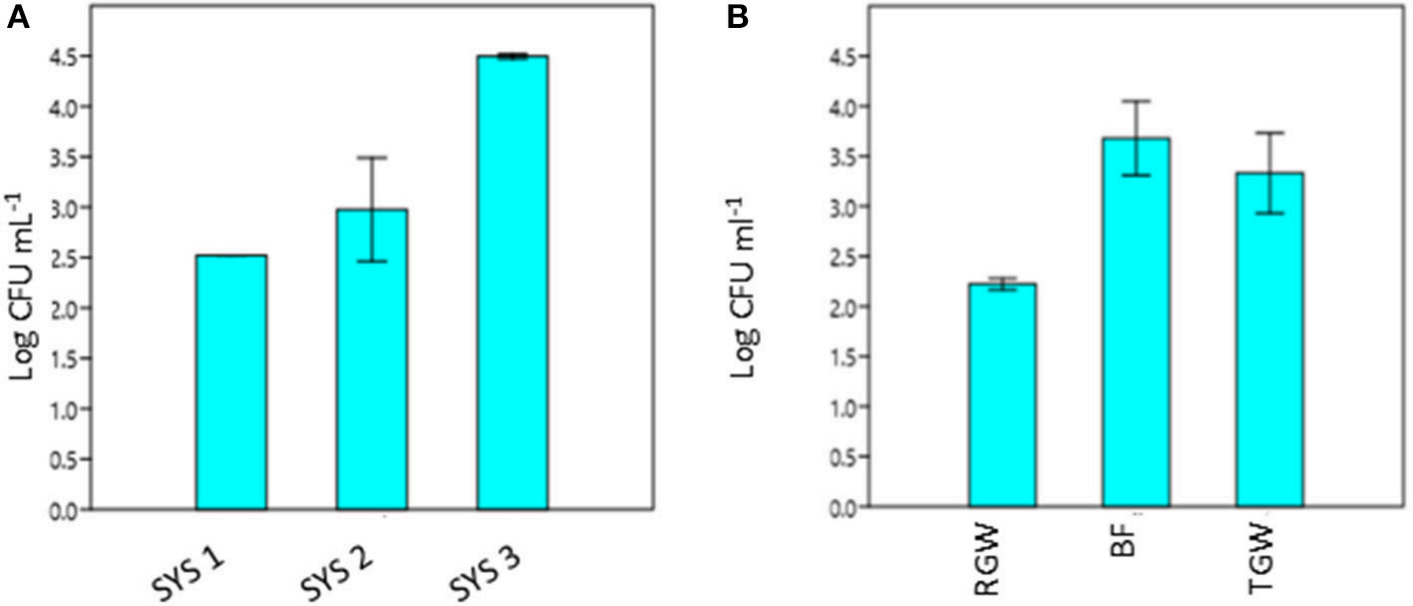
Tetracycline-resistant bacterial count (TRBC) at 25°C from treated greywater (TGW) from the three systems **(A)** and in RGW, biofilm and TGW samples from the three systems **(B)**. RGW, raw greywater; BF, biofilm; TGW, treated greywater; Log CFU mL^−1^, tetracycline-resistant bacterial count (TRBC). Error bars indicate SD of duplicate plate counts from a single system **(A)** and from each sampling point **(B)**.

The RVFCW systems were characterized by lower levels of total bacteria on the filter bed biofilm (average 8.50 × 10^4^ CFU/g^−1^) and while for SYS2, no tetracycline-resistant bacteria were detected, the SYS1 and SYS3 biofilm communities were characterized by resident tetracycline-resistant populations of between 2 and 4 Log CFU g^−1^. TGW still retained a significant level of tetracycline-resistant bacteria, since in all systems, about 1.12 × 10^4^ CFU mL^−1^ was present. Strongly significant differences (*p* < 0.01) were observed in the TRBC in TGW between the three systems, and in the SYS3 system, the highest level of tetracycline-resistant bacteria was found at 25°C (Figure 3A). Only in the SYS3 system were tetracycline-resistant bacteria isolated at both temperatures in TGW. In particular, it is possible to observe an increase in tetracycline-resistant bacteria at 25°C in treated greywater compared to raw greywater and biofilms (Figure 3B).

### Identification of tetracycline-resistant bacteria

Twenty-four species of tetracycline-resistant bacteria were identified at the genus level by 16S rDNA sequencing by Hy Laboratories Ltd. (Table 2). Even if the 16S rRNA gene was not sufficient to precisely identify a bacterial strain at the species level, we reported, in the results and the table, references to the relative species sequences that are closest to our strain. Only four of the tetracycline-resistant bacteria isolated were Gram-positive (17%), while the others were all Gram-negative (Figure S2). The isolated and characterized bacterial strains mainly belonged to the genera of *Serratia* (29%) and *Acinetobacter* (25%), while 62.2% of the isolated tetracycline-resistant bacteria belong to the class of *Gamma-Proteobacteria*.

**Table 2:** Identification of isolated tetracycline-resistant strains by 16SrDNA sequencing.

**Strain^1^**	**16s rDNA**	**GenBank acc. num**.	**Closest relative species^b^**	**GenBank acc. Num**.
SYS1-TW1	*Acinetobacter* sp.	MH090940.1	*Acinetobacter tjernbergiae*	NR117629.1
SYS1-TW2	*Acinetobacter* sp.	MH090941.1	*Acinetobacter tjernbergiae*	KM070562.1
SYS2-TW3	*Acinetobacter* sp.	MH090942.1	*Acinetobacter tjernbergiae*	KR094129.1
SYS3-TW4	*Acinetobacter* sp.	MH090943.1	*Acinetobacter junii*	AM184300.1
SYS3-TW5	*Serratia* sp.	MH090946.1	*Serratia marcescens*	CP018925.1
SYS1-RW6	*Acinetobacter* sp.	MH090944.1	*Acinetobacter junii*	AM184300.1
SYS1-RW7	*Serratia* sp.	MH090947.1	*Serratia marcescens*	CP018924.1
SYS3-RW8	*Elizabethkingia* sp.	MH090953.1	*Elizabethkingia meningoseptica*	MG982467.1
SYS3-RW9	*Serratia* sp.	MH090948.1	*Serratia marcescens*	HG738868.1
SYS1-TS10	*Achromobacter* sp.	MH090954.1	*Achromobacter insolitus*	CP026973.1
SYS3-TS11	*Lysobacter* sp.	MH090955.1	*Lysobacter enzymogenes*	AP014940.1
SYS3-US12	*Acinetobacter* sp.	MH090945.1	*Acinetobacter junii*	AM184300.1
SYS2-BF13	*Chryseobacterium* sp.	MH090956.1	*Chryseobacterium* sp.	JQ582957.1
SYS3-BF14	*Serratia* sp.	MH090949.1	*Serratia marcescens*	CP026702.1
SYS3-BF15	*Stenotrophomonas* sp.	MH090958.1	*Stenotrophomonas maltophilia*	MG982475.1
SYS3-TW16	*Serratia* sp.	MH090950.1	*Serratia marcescens*	MG982466.1
SYS3-RW17	*Serratia* sp.	MH090951.1	*Serratia marcescens*	EU048327.1
SYS3-RW18	*Serratia* sp.	MH090952.1	*Serratia marcescens*	CP026702.1
SYS1-TS19	*Chryseobacterium* sp.	MH090957.1	*Chryseobacterium lathyri*	KY933466.1
SYS1-US20	*Rummeliibacillus* sp.	MH090960.1	*Rummeliibacillus stabekisii*	CP014806.1
SYS2-BF21	*Bacillus* sp.	MH090961.1	*Bacillus cereus*	MF355368.1
SYS2-BF22	*Bacillus* sp.	MH090962.1	*Bacillus cereus*	MF355367.1
SYS2-BF23	*Bacillus* sp.	MH090963.1	*Bacillus cereus*	MF800922.1
SYS3-BF24	*Stenotrophomonas* sp.	MH090959.1	*Stenotrophomonas maltophilia*	MG982475.1

*^a^The strain ID represent the system number, the isolation location and isolate number. Raw greywater-RW, Treated grey water-TW, Biofilm on filter-BF, soil irrigated with treated grey water TS, soil irrigated with freshwater- US*.

b *All closest relative species showed 99% sequence homology*.

### Effect of chlorination on ARB survival

**Figure 4:**
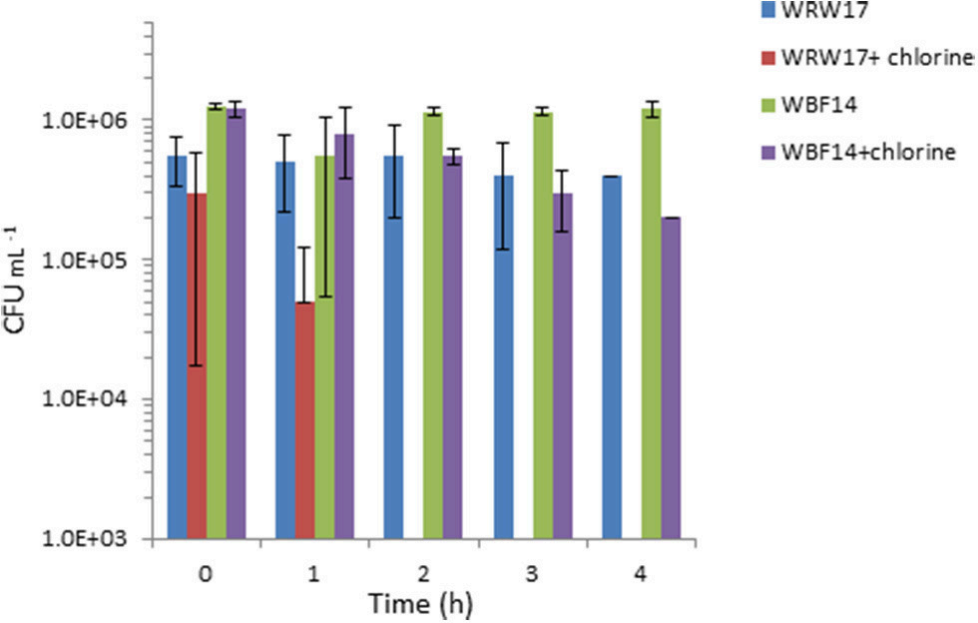
Effect of chlorination (2 mg L^−1^ NaClO) of treated greywater on the survival of two strains of isolated *Serratia* (SYS3-RW17 and SYS3-BF14). T_0_ represents the initial contact time with the chlorine. After 2 h, WRW17+chlorine counts were “0”.

We selected two *Serratia* strains with high tetracycline resistance as indicators for the chlorination effectiveness of the TGW. The results show that the two strains of *Serratia* isolated from RGW and BF (Figure 4) were able to survive despite having been exposed to a high concentration of chlorine (2 mg L^−1^). In particular, the growth of the *Serratia* strain isolated from RGW at 37°C was inhibited after the second hour, while the *Serratia* strain isolated from the biofilm samples at 25°C decreased only by 90% (10% survival) after 4 h in comparison to the non-chlorinated control.

### Minimum inhibition concentration (MIC)

The MIC's results were compared with the epidemiological cut-off values for resistance (ECOFFs) established by EUCAST: all the microorganism with acquired resistance showed higher MIC values than the epidemiological cut-off value, so according to EUCAST all the microorganism were very resistant to antibiotics (http://www.eucast.org/mic_distributions_and_ecoffs). To highlight the different resistance levels of isolated microorganisms, according to the obtained MIC values (μg mL-1), five different resistance levels were identified for the isolated strains, as follows: sensitive (S): MIC values between 0 and 50 μg mL^−1^; low resistance (L): MIC values between 50 and 100 μg mL^−1^; medium resistance (M): MIC values between 150 and 350 μg mL^−1^; high resistance (H): MIC values between 350 and 500 μg mL^−1^; and very high resistance (VH): MIC values higher than 500 μg mL^−1^. Based on this classification, most of the tetracycline-resistant bacteria isolated were considered to have medium resistance (Table 3). Only one *Serratia* strain (TW5) isolated from the TGW of SYS3 had a lower resistance (L).

**Table 3:** Minimum inhibitory concentration (MIC) of the tested antibiotics on the tetracycline-resistant bacterial strain.

			**MIC (ug** ***mL***^**−1**^**)**	
**Strain**	**Microrganism^a^**	**Tet**	**Amox**	**Kana**	**Cipro**
SYS1-TW1	*Acinetobacter tjembergiae*	300	S	S	S
SYS1-TW2	*Acinetobacter tjembergiae*	250	S	S	S
SYS2-TW3	*Acinetobacter tjembergiae*	250	150	S	S
SYS3-TW4	*Acinetobacter junii*	≥500	S	S	S
SYS3-TW5	*Serratia marcescens*	50	150	≤50	100
SYS1-RW6	*Acinetobacter junii*	250	S	≤50	≤50
SYS1-RW7	*Serratia marcescens*	400	≤50	≤50	≤50
SYS3-RW8	*Elizabethkingia endophytica*	150	≥300	≥300	≤50
SYS3-RW9	*Serratia marcescens*	≥500	80	100	≤50
SYS1-TS10	*Achromobacter insolitus*	250	150	200	S
SYS3-TS11	*Lysobacter enzymogenes*	≥500	≥300	200	≤50
SYS3-US12	*Acinetobacter junii*	≥500	S	S	S
SYS2-BF13	*Chryseobacterium* sp.	300	≥300	≥300	S
SYS3-BF14	*Serratia marcescens*	300	S	150	≤50
SYS3-BF15	*Stenotrophomonas maltophilia*	≥500	S	200	≤50
SYS3-TW16	*Serratia marcescens*	≥350	150	S	S
SYS3-RW17	*Serratia marcescens*	≥350	50	S	S
SYS3-RW18	*Serratia marcescens*	350	150	<50	S
SYS1-TS19	*Chryseobacterium lathyri*	≥350	S	150	100
SYS1-US20	*Rummeliibacillus stabekisii*	300	S	S	S
SYS2-BF21	*Bacillus cereus*	300	S	S	S
SYS2-BF22	*Bacillus cereus*	350	S	S	S
SYS2-BF23	*Bacillus cereus*	300	S	S	S
SYS3-BF24	*Stenotrophomonas maltophilia*	300	150	S	S

a *The names refer to the closest relative species identified by 16SrDNA sequencing (Table 2)*.

*Tet, Tetracycline; Amox, Amoxicillin; Kana, Kanamycin; Cipro, Ciprofloxacin*.

*≤ = microorganism growth was inhibited by the lowest concentration of the antibiotic tested*.

*≥ = microorganism growth was not inhibited by the higher concentration of antibiotic tested*.

*Green, Sensitive; Yellow, Lower (<50 to 100 μg mL^-1^); Orange, Medium (from 150 to 350 μg mL^-1^); Red, High (from 350 to 500 μg mL^-1^); Dark red, Very High (≥500 μg mL^-1^)*.

Five strains (SYS3-TW4, SYS3-RW9, SYS3-TS11, SYS3-US12, and SYS3-BF15) were able to grow at higher tetracycline concentrations than those tested (≥ 500 μg/ml), so they have a very high resistance (VH). Five strains (SYS3-TW5, SYS1-RW7, SYS3-RW8, TWRW9, and SYS3-TS11) were also shown to be resistant to the other three tested antibiotics. It has been observed that tetracycline-resistant bacteria were more sensitive to ciprofloxacin than to amoxicillin and kanamycin. Nine tetracycline-resistant bacteria (SYS2-TW3, SYS1-RW6, SYS3-RW8, SYS1-TS10, SYS3-S11, SYS2-BF13, SYS3-TW16, SYS3-RW18, and SYS3-BF24) showed a medium resistance to amoxicillin, and two (SYS3-RW8 and SYS3-TS11) of them were also able to survive at higher concentrations of kanamycin (higher than 300 mg L^−1^).

### Tetracycline resistance gene characterization

Based on the PCR analysis, none of the isolated resistant strains were positive for the *tet*A or *tet*B (efflux) or for the *tet*M, *tet*Q, or *tet*W (ribosomal) genes (Table 4). It was found that 58% of the tetracycline-resistant isolates were positive for *tet*39, all isolated at 25°C. All the tetracycline-resistant bacteria were also positive for at least one of the β-lactamase genes tested. In particular, 79% were positive for blaTEM, 58% were positive for blaCXTM-32, 67% were positive for blaOXA-2, 12.5% for blaOXA-10 and only 8% for blaSHV.

**Table 4:** Occurrence of tetracycline and β-lactamase resistance genes in the bacterial isolates, determined by PCR.

				**Beta-lactamase**		**Tetracycline**^**b**^	
**Strain**	**Microorganism^a^**	**blaTEM**	**BlaCTM_32**	**blaSHV**	**blaOXA-2**	**blaOXA-10**	**Tet(39)**
SYS1-TW1	*Acinetobacter tjembergiae*	+	–	–	–	–	+
SYS1-TW2	*Acinetobacter tjembergiae*	+	–	–	+	–	+
SYS2-TW3	*Acinetobacter tjembergiae*	+	+	–	–	–	+
SYS3-TW4	*Acinetobacter junii*	+	–	–	+	–	+
SYS3-TW5	*Serratia marcescens*	+	–	–	–	–	+
SYS1-RW6	*Acinetobacter junii*	+	+	–	+	–	+
SYS1-RW7	*Serratia marcescens*	–	–	–	–	–	+
SYS3-RW8	*Elizabethkingia endophytica*	+	+	–	+	–	+
SYS3-RW9	*Serratia marcescens*	+	–	–	+	–	+
SYS1-TS10	*Achromobacter insolitus*	+	+	–	+	–	+
SYS3-TS11	*Lysobacter enzymogenes*	+	+	–	+	–	–
SYS3-US12	*Acinetobacter junii*	+	–	–	+	+	+
SYS2-BF13	*Chryseobacterium* sp.	+	–	+	–	–	+
SYS3-BF14	*Serratia marcescens*	+	+	+	+	–	+
SYS3-BF15	*Stenotrophomonas maltophilia*	+	+	–	+	–	+
SYS3-TW16	*Serratia marcescens*	–	–	–	+	–	–
SYS3-RW17	*Serratia marcescens*	+	+	–	+	–	–
SYS3-RW18	*Serratia marcescens*	+	+	–	+	–	–
SYS1-TS19	*Chryseobacterium lathyri*	–	+	–	+	–	–
SYS1-US20	*Rummeliibacillus stabekisii*	+	+	–	+	–	–
SYS2-BF21	*Bacillus cereus*	+	+	–	+	–	–
SYS2-BF22	*Bacillus cereus*	–	–	–	–	–	–
SYS2-BF23	*Bacillus cereus*	+	+	–	–	+	–
SYS3-BF24	*Stenotrophomonas maltophilia*	–	+	–	–	+	–

a *The names refer to the closest relative species identified by 16SrDNA sequencing (Table 1)*.

b *Only tet39 is reported among the six tet genes examined, since the strains were all negative for the other five genes*.

### Sequencing of the *tet*39 gene

**Figure 5:**
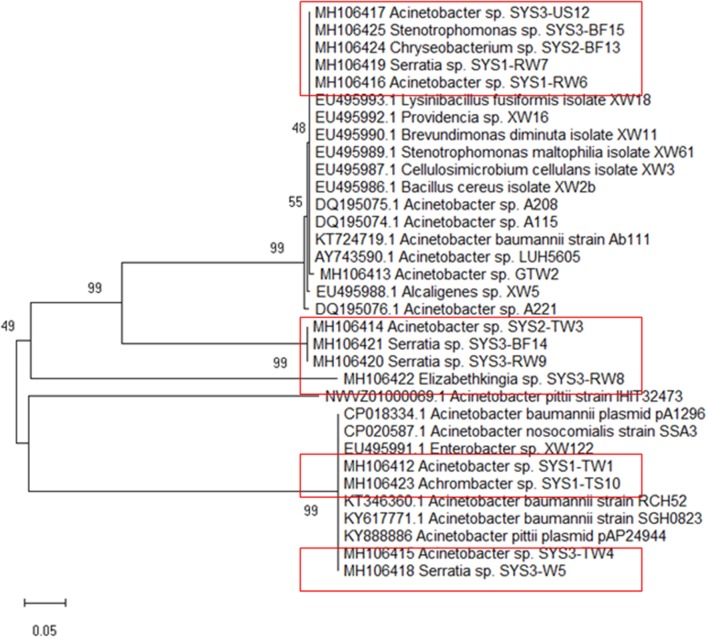
Phylogenetic tree of *tet*39 genes sequenced from the positive strains (in box) was inferred using the Neighbor-Joining method. The percentage of replicate trees in which the associated taxa clustered together in the bootstrap test (500 replicates) are shown next to the branches (Kumar et al., 2016).

Because *tet*39 (conferring resistance via an active efflux pump) was found to be the most abundant resistance gene determinant, its PCR amplicons were sequenced for a better understanding. The results revealed that two different genotypes belonging to two clusters (cluster A and cluster B) were randomly observed among *tet*39 resistance bacteria (Figure 5). In both clusters, the *tet*39 sequences were very similar across different genera.

## Discussion

The need to treat greywater before reuse at a local scale led to the development of a small on-site RVFCW bioreactor that is effective in removing chemical and biological contaminants (see Table S2) (Gross et al., 2007). However, the possible risk of spreading opportunistic pathogens after the treatment was also considered (Benami et al., 2016). Because of the proximity between the treatment units and the point of greywater reuse, it is also important to investigate other microbiological factors such as antibiotic resistance in the treated greywater's microbial community.

The presence of antibiotic-resistant genes (ARGs), such as tetracycline and beta-lactam resistance genes, have been reported in wastewater (Szczepanowski et al., 2009; Karkman et al., 2017; Voolaid et al., 2018), but the current study represents the first investigation on ARB in greywater. Previous studies on ARB in municipal wastewater (Huang et al., 2012; Harnisz et al., 2015) reported tetracycline-resistant bacterial levels in the range of 10^2^-10^3^ CFU mL^−1^, and their findings are consistent with our present results for treated greywater (TGW) in the SYS1 and SYS2 systems at 25°C. In SYS3 system, the tetracycline-resistant bacterial count (TRBC) was higher than in SYS1 and SYS2 systems (Figure 3A). These differences may be related to many factors such as health status of inhabitants, age, number or lifestyles. Our study, however, did not examine these parameters, so the origin of the TRBC remains uncertain. Our study also examined whether the irrigation caused a buildup of resistance in the soil; it is worth noting that a significant difference in the TRBC was not observed (*p* > 0.05) in the freshwater and treated greywater irrigated soils (Figure 1). These findings are in agreement with previous studies which demonstrated that irrigation with wastewater does not seem to impact antibiotic resistance levels in the soil microbiome (Gatica and Cytryn, 2013). However, we cannot exclude the greywater as a possible source of the tetracycline-resistant bacteria even if the existence of tetracycline-resistant strains in both treated and untreated soil could prove that the greywater is not the only contamination source. We also need to consider the possibility of a cross- or direct contamination caused by humans or animals that could contribute to the spread of ARB, bypassing the irrigation water route. A recent publication regarding the contribution of treated effluents to the soil resistome stated that while antibiotic resistance levels in soil are increased temporally by land application of wastes, their persistence is not guaranteed and is, in fact, variable, and often contradictory, depending on the application site (Pepper et al., 2018).

In all three systems, we observed a significant (*p* < 0.05) increase in the TRBC at 25°C in BF and in TGW compared to RGW (Figure 3B). Bacteria retained inside the filters could be the explanation for this observation. The RVFCW can be considered as a biofilm-based wastewater treatment system such as a trickling filter wastewater treatment. Balcázar et al. (2015) proposed, based on many studies, that environmental biofilms are true reservoirs of ARGs. Thus, a concentration effect within the system is a possible explanation for the presence of tetracycline-resistant bacteria in the treated water. In contrast, however, a recent study that compared abundances of ARGs in activated sludge and a trickling filter suggested that there is no difference in the prevalence of ARG mobilization in the treated effluents (Petrovich et al., 2018).

In our study, it was not possible to make a comparison at 37°C between the TRBCs in the three systems, since at 37°C, the tetracycline-resistant bacteria were isolated only in the number three system. We hypothesize that the resistant bacteria that grow best at 37°C represent enteric or fecal microorganisms. The low detection of these bacteria agrees with the effective removal of fecal coliform to the level of 2 CFU per 100 ml in the examined RVFCW after disinfection (Benami et al., 2016). Our results suggest that these three systems were unable to prevent ARB survival after greywater treatment, and to achieve this goal, additional treatment methods need to be included, such as the use of chlorine or UV disinfection. Our results showed that chlorination was effective in immediately inactivating three out of five tested isolated *Serratia* strains, so ARB removal could be a possible solution, even if some strains can survive a longer contact time (Figure 3). As reported in the literature, conflicting results still exist concerning ARB removal by chlorination (Yuan et al., 2015). Some researchers reported effective ARB reduction using this method (Huang et al., 2011), whereas other results indicated that chlorination did not significantly reduce ARB (Munir et al., 2011).

The isolated and characterized bacterial strains belonged mainly to the genera *Serratia* sp. (29%) and *Acinetobacter* sp. (25%). As previously mentioned, *Acinetobacter* sp. is a particularly suitable genus for monitoring antibiotic resistance in the environment; in fact, until recently, bacterial screening of WWTP influents and effluents usually focused on *Acinetobacter* spp. (Zhang Y. et al., 2009; Voolaid et al., 2018). Similarly to our case, in a previous study on treated wastewater, tetracycline-resistant strains of *Serratia marcescens* and *Acinetobacter spp*. were isolated (Harnisz et al., 2015). In that case, the simultaneous presence of two resistance determinants, *tet*(A) and *tet*(B), was documented, while in our experiment, all the tetracycline-resistant strains were negative for these two genes. It must be noted that a fecal indicator bacteria survey was not conducted since other authors already tested the same systems for this purpose (Benami et al., 2013).

According to our classification, 62.5% of the bacteria showed at least a medium resistance to tetracycline. It should be mentioned that according to EUCAST's epidemiological cut-off values for AR (ECOFFs) (The European Committee on Antimicrobial Susceptibility Testing, 2018) MIC levels above >100 g mL^−1^ are already considered a high resistance trait. Thus, the possible spread of high dose antibiotic resistance determinants in the environment in which greywater is used for irrigation is worthy of concern.

In this study, tetracycline-resistant bacteria were positive only for *tet*39, and this confirmed the fact that even if *tet*39 remains closely associated with *Acinetobacter* spp., its plasmid location should enable dissemination to other species (Coyne et al., 2011). Initially, it was thought that the *tet*39 gene was one of the efflux genes unique to environmental bacteria (Roberts, 2011), but then it was understood that this gene, associated with mobile elements, could be transferred back and forth between environmental and non-environmental bacteria.

We focused our work on six tetracycline resistance genes from the 46 genes currently known. They were the *tet*A, *tet*B, and *tet*39 (efflux), and *tet*M, *tet*Q and *tet*W (ribosomal) genes that are the most common tetracycline resistance genes among Gram-negatives (Zhang Y. et al., 2009; Roberts, 2011; Roberts et al., 2015a). The search of all the genes that determine the tetracycline resistance in the isolated bacteria falls beyond the scope of the manuscript. Other surveys of tetracycline resistance genes focused on covering the most common genes (Henriques et al., 2008; Nikolakopoulou et al., 2008; Tao et al., 2010; Harnisz et al., 2015).

We also need to consider that the Gram-negative efflux genes are widely distributed and generally associated with large plasmids, most of which are conjugative, which often carry other antibiotic resistance genes. This phenomenon contributed to the dramatic increase of the multiple-drug-resistant bacteria over the last 40 years (Chopra and Roberts, 2001). Many of the tetracycline efflux resistance genes are found mostly in environmental strains but can also be found in bacteria associated with humans and animals (Roberts, 2011).

It is noteworthy that the majority of the isolated strains shared *tet*39, independent of the source (water, biofilm, and soil) and the location (SYS1, 2, and 3), even if we cannot exclude the possibility that isolated strains may harbor other tetracycline resistance genes. It has been reported that both Gram-positives and more than 10% of Gram-negatives could carry multiple tetracycline resistance genes (Roberts, 2005); thus it is essential to specify that the different tetracycline genes can have either the same mode of action (efflux or ribosomal protection) or different modes of action (efflux and ribosomal protection), as do the pathogenic and opportunistic species (Chopra and Roberts, 2001).

All the isolated *tet*39 resistance bacteria were positive for at least one of the β-lactamase genes tested. In fact, the majority of the 30 tetracycline resistance efflux genes are usually associated with plasmids (Roberts, 2005) that often carry other antibiotic resistance genes (such as those that confer aminoglycoside, β-lactam resistance), heavy metal resistance genes, or pathogenic factors such as toxins (Chopra and Roberts, 2001). Therefore, it indicates the increasing possibility of multidrug resistance and environmental dissemination.

Except for two isolates, beta-lactam resistance genes have been found in all amoxicillin-susceptible bacteria, confirming the fact that even low concentrations of antibiotics can result in the selection of ARGs. This makes it very difficult to establish a safe concentration of an antibiotic compound in wastewater (Karkman et al., 2017).

The isolated strains showed *tet*39 sequences belonging to two different genotypes separated in two distinct clusters (A and B) by cluster analysis (Figure 4). It must be highlighted that the genotype associated with cluster A in this study has been identified in bacteria belonging to different genera (*Bacillus* sp., *Acinetobacter* sp.*, Stenotrophomonas* sp.) isolated from environmental samples, mostly of aquatic origin (Agersø and Guardabassi, 2005; Adelowo and Fagade, 2009; Roberts and Schwarz, 2015b; Hamidian et al., 2016). Additionally, the genotype associated with cluster B in this study has been identified in bacteria isolated from clinical samples such as blood or sputum Adelowo and Fagade, 2009; Hamidian et al., 2016; Yoon et al., 2017). In our study, *tet*39 resistance bacteria harbored both genotypes (environmental and clinical), independent of source and isolation temperature. This observation demonstrates the possible transfer of the *tet*39 gene between bacteria of clinical and environmental origin.

It is interesting to note that the *tet*39 gene from bacteria of clinical origin was not present among the samples isolated from the SYS3, but given the limited number of samples, we cannot be entirely confident of its absence.

Among the *tet*39 resistance bacteria belonging to the two different clusters, there were no significant differences between the tetracycline's MIC values. This is most likely due to the fact that other genes are involved in the resistance to tetracycline; indeed, as previously noted, all Gram-positives and more than 10% of Gram-negatives could carry multiple tetracycline genes (Roberts, 2005).

## Conclusions

The ARB isolated in this study were not obligatory pathogens. The fact that *tet*39 was the dominant resistance gene may arise from its broad host range. Like other biological wastewater treatment systems, the RVFCW system does not remove all of the ARB present in the raw greywater. Most likely, the filter bed biofilm of the system contributed to the ARB community in the treated effluents. Thus, additional treatment methods such as chlorination need to be included in this system to minimize the ARB numbers in the effluent. Interestingly, the ARB abundance in the TGW-irrigated soil and the freshwater-irrigated soil did not alter, suggesting that ARB did not accumulate in the TGW-irrigated soil.

To safely eliminate ARB from greywater, further studies should be carried out to understand how the transfer of ARGs occurs. Of particular importance is the determination of whether specific compounds abundant in greywater (e.g., detergents) lead to resistance evolution. This work dealt with the detection of Tetracycline-resistant bacteria and Tetracycline resistant genes on greywater, in the system and the soil also evaluating the potential multiple resistance. Preliminary genetic relations between the tet39 genes isolated showed a possible exchange between clinical and environmental strains. However, further study needs to be done to understand the clonal relations between the isolates better understand the clonal relations between the isolates and strengthen our results.

## Author contributions

ET performed the experiments, analyzed the data, and wrote a draft manuscript. LB analyzed the data and supervised the writing of the draft manuscript. AG aided in interpreting the results and worked on the manuscript. ZR conceived the study and was in charge of the overall direction and planning, as well as writing the manuscript.

### Conflict of interest statement

The authors declare that the research was conducted in the absence of any commercial or financial relationships that could be construed as a potential conflict of interest.
