# Supplementary material for: Antibiotic-Resistant Bacteria in Greywater and Greywater-Irrigated Soils
Source: Front Microbiol. 2018 Nov 6;9:2666. doi: 10.3389/fmicb.2018.02666 (PMC6232274; doi:10.3389/fmicb.2018.02666)
Supplement: Supplementary file 1 [file Data_Sheet_1.docx]

**ANTIBIOTIC-RESISTANT BACTERIA IN GREYWATER AND GREYWATER-IRRIGATED SOILS**

Eleonora Troiano^1^, Luciano Beneduce^1^, Amit Gross^2^ and Zeev Ronen^2*^

^1^Department of the Sciences of Agriculture, Food and Environment, University of Foggia, Via Napoli 25, 71122 Foggia, Italy

^2^Department of Environmental Hydrology and Microbiology, Zuckerberg Institute for Water Research, Jacob Blaustein Institutes for Desert Research, Ben-Gurion University of the Negev, Sede Boqer Campus, 84990 Midreshet Ben-Gurion, Israel

**Supplementary data**

Table S1: Physical chemical characteristics of raw and treated greywater in the three systems over the research period.

| Parameter | Raw | Treated | Israeli standard for  irrigation of treated wastewater (Inbar, 2007) |
| --- | --- | --- | --- |
| pH | 8.0±0.8 | 8.1±0.4 | 6.5-8.5 |
| EC | 0.9±0.3 | 0.8±0.2 | 1.8 |
| Turbidity (NTU) | 57±45 | 6±11 | NA |
| TSS (mg/l) | 77±56 | 9.4±10 | 15 |
| COD (mg/l) | 209±202 | 34±28 | 150 |
| BOD5 (mg/l) | 136±121 | 3.9±4.5 | 15 |
| TN (mg/l) | 13±9.4 | 7.2±7.3 | 35 |
| TP (mg/l) | 2.1±1.8 | 0.6±0.8 | 7 |
| Boron | 0.43±0.1 | 0.46±0.1 | 0.5 |
| Surfactants | 4.6±6.9 | 0.44±0.2 | 3 |

Table S2: PCR reaction and thermal profiles for ARG detection in the isolated strains.

| Target gene | Reaction Mixture | Volumes (µl) | Thermal profile |
| --- | --- | --- | --- |
|  |  |  |  |
|  | 10 x PCR reaction buffer | 2,5 µl |  |
|  | MgCl_2_ (50mM) | 1 µl | 95°C for 5 min |
|  | dNTPs (100 mM) | 0,5µl | 95°C for 15 sec 35 x |
| blaTEM | BIO-X-ACT Short (BIOLINE) | 0,5µl | 60°C for 30 sec 35 x |
|  | qblaTEM- F (FW) 10 µM | 1,25 µl | 72°C for 1 min 35 x |
|  | qblaTEM-R (RV) 10 µM | 1,25µl | 72°C for 5 min |
|  | Template DNA | 2 µl |  |
|  | Water | 16µl |  |
|  | 10 x PCR reaction buffer | 2,5 µl |  |
|  | MgCl_2_ (50mM) | 1 µl | 95°C for 5 min |
|  | dNTPs (100 mM) | 0,5µl | 95°C for 30 sec 35 x |
| blaSHV | BIO-X-ACT Short (BIOLINE) | 0,5µl | 64°C for 30 sec 35 x |
|  | bla-SHV- FW 10 µM | 0,5 µl | 72°C for 30 sec 35 x |
|  | bla-SHV-RV 10 µM | 0,5µl | 72°C for 10 min |
|  | Template DNA | 2,5µl |  |
|  | Water | 17µl |  |
|  | 10 x PCR reaction buffer | 2,5 µl |  |
|  | MgCl_2_ (50mM) | 1 µl | 95°C for 5 min |
|  | dNTPs (100 mM) | 0,5µl | 95°C for 15 sec 35 x |
| blaCTXM-32 | BIO-X-ACT Short (BIOLINE) | 0,5µl | 68°C for 30 sec 35 x |
|  | CTXM-FW 10 µM | 1,25 µl | 72°C for 1 min 35 x |
|  | CTXM-RV 10 µM | 1,25µl | 72°C for 5 min |
|  | Template DNA | 2 µl |  |
|  | Water | 16µl |  |
|  | 10 x PCR reaction buffer | 2,5 µl |  |
|  | MgCl_2_ (50mM) | 1 µl | 95°C for 5 min |
|  | dNTPs (100 mM) | 0,5µl | 95°C for 15 sec 35 x |
| blaOXA-2 | BIO-X-ACT Short (BIOLINE) | 0,5µl | 60°C for 30 sec 35 x |
|  | OXA-2 FW 10 µM | 1,25 µl | 72°C for 40 sec 35 x |
|  | OXA-2 RV 10 µM | 1,25µl | 72°C for 5 min |
|  | Template DNA | 2 µl |  |
|  | Water | 16µl |  |
|  | 10 x PCR reaction buffer | 2,5 µl |  |
|  | MgCl_2_ (50mM) | 1 µl | 95°C for 5 min |
|  | dNTPs (100 mM) | 0,5µl | 95°C for 15 sec 35 x |
| blaOXA-10 | BIO-X-ACT Short (BIOLINE) | 0,5µl | 60°C for 30 sec 35 x |
|  | OXA-10 FW 10 µM | 1,25 µl | 72°C for 1 min 35 x |
|  | OXA-10 RV 010 µM | 1,25µl | 72°C for 5 min |
|  | Template DNA | 2 µl |  |
|  | Water | 16µl |  |
|  | 10 x PCR reaction buffer | 2,5 µl |  |
|  | MgCl_2_ (50mM) | 1 µl | 95°C for 5 min |
|  | dNTPs (100 mM) | 0,5µl | 95°C for 15 sec 35 x |
| TetM | BIO-X-ACT Short (BIOLINE) | 0,5µl | 55°C for 30 sec 35 x |
|  | TetM- FW 10 µM | 1 µl | 72°C for 40 sec 35 x |
|  | TetM-RV 10 µM | 1 µl | 72°C for 5 min |
|  | Template DNA | 2,5µl |  |
|  | Water | 16 µl |  |

Continue - Table S2: PCR reaction and thermal profiles for ARG detection in the isolated strains.

| Target gene | Reaction Mixture | Volumes (µl) | Thermal profile | | |  |
| --- | --- | --- | --- | --- | --- | --- |
|  | 10 x PCR reaction buffer | 2,5 µl |  | | |  |
|  | MgCl_2_ (50mM) | 1 µl | 95°C for 5 min | | |  |
|  | dNTPs (100 mM) | 0,5µl | 95°C for 15 sec 35 x | | |  |
| TetQ | BIO-X-ACT Short (BIOLINE) | 0,5µl | 63°C for 30 sec 35 x | | |  |
|  | TetQ- FW 10 µM | 1 µl | 72°C for 40 sec 35 x | | |  |
|  | TetQ -RV 010 µM | 1 µl | 72°C for 5 min | | |  |
|  | Template DNA | 2,5µl |  | | |  |
|  | Water | 16 µl |  | | |  |
|  | 10 x PCR reaction buffer | 2,5 µl |  | | |  |
|  | MgCl_2_ (50mM) | 1 µl | 95°C for 5 min | | |  |
|  | dNTPs (100 mM) | 0,5µl | 95°C for 15 sec 35 x | | |  |
| TetW | BIO-X-ACT Short (BIOLINE) | 0,5µl | 64°C for 30 sec 35 x | | |  |
|  | TetW- FW 10 µM | 1 µl | 72°C for 40 sec 35 x | | |  |
|  | TetW-RV 10 µM | 1 µl | 72°C for 5 min | | |  |
|  | Template DNA | 2,5µl |  | | |  |
|  | Water | 16 µl |  | | |  |
|  | 10 x Buffer | 2,5µl | | |  |  |
|  | dNTPs (0,2mM) | 0,5 µl | | | 95°C for 3 min |  |
| tet(A) | DreamTaq DNA Polymerase (Thermo Scientific) (1,25U in 50µl) | 0,25 µl | | | 95°C for 45 sec 35 x |  |
|  | Tet A-FW 10 µM | 1 µl | | | 57°C for 30 sec 35 x |  |
|  | TetA- RV 10 µM | 1 µl | | | 72°C for 1 min 35 x |  |
|  | Template DNA | 2 µl | | | 72°C for 5 min |  |
|  | Water | 17,75µl | | |  |  |
|  | 10 x Buffer | 2,5µl | |  | | |
|  | dNTPs (0,2mM) | 0,5 µl | | 95°C for 3 min | | |
| tet(B) | DreamTaq DNA Polymerase (Thermo Scientific) (1,25U in 50µl) | 0,25 µl | | 95°C for 45 sec 35 x | | |
|  | Tet B-FW 10 µM | 1 µl | | 50°C for 30 sec 35 x | | |
|  | TetB- RV 10 µM | 1 µl | | 72°C for 1 min 35 x | | |
|  | Template DNA | 2 µl | | 72°C for 5 min | | |
|  | Water | 17,75µl | |  | | |
|  | 10 x Buffer | 2,5µl | |  | | |
|  | dNTPs (0,2mM) | 0,5 µl | | 95°C for 3 min | | |
| tet(39) | DreamTaq DNA Polymerase (Thermo Scientific) (1,25U in 50µl) | 0,25 µl | | 95°C for 45 sec 35 x | | |
|  | tet(39)-FW 10 µM | 1 µl | | 50°C for 30 sec 35 x | | |
|  | tet(39)- RV 10 µM | 1 µl | | 72°C for 1 min 35 x | | |
|  | Template DNA | 2 µl | | 72°C for 5 min | | |
|  | Water | 17,75µl | |  | | |


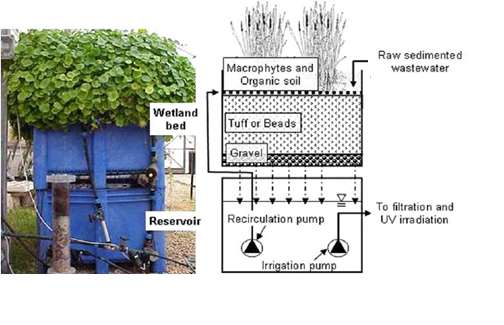
Figure S1. A schematic description of the recirculating vertical flow constructed wetland (RVFCW) right, and a picture of actual system on the left. The system is operated at the hydraulic retention time of 7h with a circulation rate of 1000 litter per hour. The loading rate, depending on the household site, was between 300-500 litters per day. The porosity of the filter is 40%, and it remained constant (no clogging). (After Gross et al., 2008).

Gram + (17%)

Figure S2: Tet resistance distribution in the different bacteria genus isolated.

References

Gross, A., Sklarz, M. Y., Yakirevich. A., & Soares, M. I. M. (2008). Small scale recirculating vertical flow constructed wetland (RVFCW) for the treatment and reuse of wastewater. *Water Sci. Technol.* 58, 487-494. https://doi.org/10.2166/wst.2008.367

Inbar Y. (2007). New standards for treated wastewater reuse in Israel. In Wastewater reuse–Risk assessment, decision-making and environmental security, 291-296. Springer, Dordrecht. https://doi.org/10.1007/978-1-4020-6027-4_28
